# Human pluripotent stem cell-derived DDX4 and KRT-8 positive cells participate in ovarian follicle-like structure formation

**DOI:** 10.1016/j.isci.2020.102003

**Published:** 2020-12-29

**Authors:** Danny C.W. Yu, Fang-Chun Wu, Chia-Eng Wu, Lu-Ping Chow, Hong-Nerng Ho, Hsin-Fu Chen

**Affiliations:** 1 Department of Obstetrics and Gynecology, College of Medicine and the Hospital, National Taiwan University, Taipei, Taiwan; 2 Graduate Institute of Medical Genomics and Proteomics, College of Medicine, National Taiwan University, Taipei, Taiwan; 3 Institute of Immunotherapy, Fujian Medical University, Fujian, China; 4 Aging and Disease Prevention Research Center, and Department of Medical Laboratory Sciences and Biotechnology, Fooyin University, Kaohsiung, Taiwan; 5 Graduate Institute of Immunology, College of Medicine, National Taiwan University, Taipei, Taiwan; 6 Graduate Institute of Biochemistry and Molecular Biology, College of Medicine, National Taiwan University, Taipei, Taiwan

**Keywords:** Molecular Biology, Cell Biology, Stem Cell Research, Developmental Biology

## Abstract

Understanding the mechanisms of human pluripotent stem cells (hPSCs) specification, development and differentiation to gametes are useful for elucidating the causes of infertility and potential treatment. This study aims to examine whether hPSCs can be induced to DDX4 extracellularly expressing primordial germ cell-like cells (DDX4^ec^ PGCLCs) and further into ovarian follicle stage in a combined *in vitro* and *in vivo* model. The transcriptional signatures show that these DDX4^ec^ PGCLCs are characteristic of PGCs and express ovarian folliculogenesis markers. We also verify that keratin (KRT)-8 is highly expressed in the DDX4^ec^ PGCLCs and plays a crucial role in germ cell migration. By co-culturing DDX4^ec^ PGCLCs with human granulosa cells (GCs), these cells are further induced into ovarian follicle-like structures in a xenograft mice model. This approach can in the future design practical strategies for treating germ cell-associated issues of infertility.

## Highlights

• hPSC-derived DDX4 PGCLCs participate ovarian follicle-like structure formation
• Human granulosa cells as a niche environment are participating folliculogenesis
• Keratin 8 plays an essential role in primordial germ cell migration

## Graphical abstract

**Figure:**
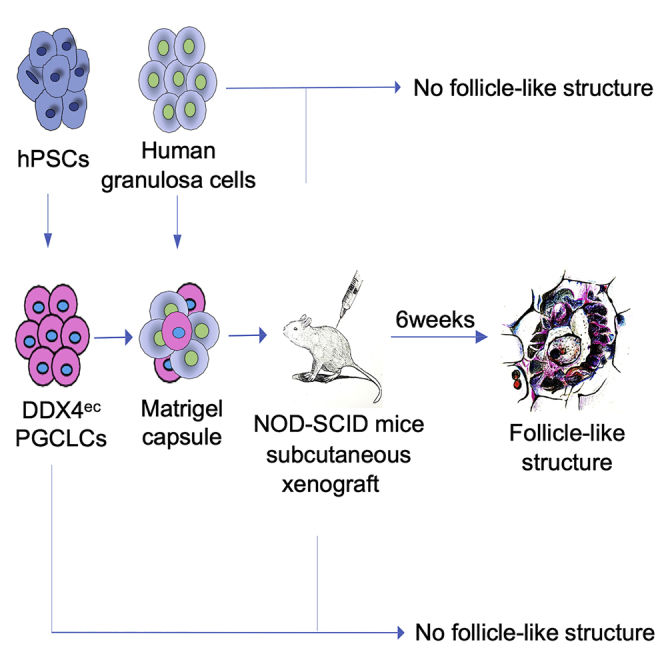

## Introduction

Germ cells are critical players in human reproduction. In modern infertility treatment, the quality and quantity of oocytes remain the major determinants of successful outcomes. However, the female reproductive potential is limited by the restricted number of eggs available in the ovaries and the quality of eggs that decline with age. For infertile patients with premature ovarian failure or due simply to ovarian aging, the reduced quality of eggs in the ovaries is often the primary reason leading to the subfertility. It is thus useful to explore efficient techniques to derive human gametes for the purpose of disease modeling to improve egg number and quality, or hopefully be used as a direct application to assisted reproduction. Human pluripotent stem cells (hPSCs) including embryonic stem cells (ESCs) and induced pluripotent stem cells (iPSCs) have the potential to develop into all cell types including germ cells in human. Although functional oocytes from mouse ESCs (mESCs) and iPSCs (miPSCs) have successfully been derived (Hayashi et al., 2011, 2012), mature human gametes have not yet been established from stem cells, except one recent report by Yamashiro et al., who reported the generation of human oogonia and immediate precursory states for oocytes from hiPSCs in culture (Yamashiro et al., 2020). Up to now, our knowledge of human mammalian primordial germ cell (PGC) specification and epigenetic reprogramming remains imprecise. Knowledge about mammalian PGC specification was initially established in mice (Saitou et al., 2002; von Meyenn et al., 2016), specified from post-implantation epiblast cells on embryonic day (E) 6.25. Subsequently, PGCs migrate through the hindgut to the developing genital ridges in response to bone morphogenetic protein 4 (BMP4) pathway signaling before sexual differentiation (Lawson et al., 1999; Saitou and Miyauchi, 2016). Evidence shows that the treatment with multiple cytokines and aggregated with male or female gonadal somatic cells leads to the differentiation of mESCs and miPSCs toward epiblast-like cells (EpiLCs), to PGC-like cells (PGCLCs), as well as the spermatogenesis and oogenesis (Hayashi et al., 2012; Hikabe et al., 2016; Kimura et al., 2014). However, significant differences exist in germ cell specification between mouse and human such as the developmental timeline, efficiency, signaling pathways, transcriptional, translational, and metabolic properties of developmentally similar cell types (Yamashiro et al., 2020). We thus need to explore the differences and identify the ways of deriving germ cells from human PSCs.

Previous studies have made a significant improvements toward the identification and characterization of PGCs in mice and human by the markers such as PRDM14 (Yamaji et al., 2008), cell surface makers KIT (Gkountela et al., 2015; Guo et al., 2015), CD38 (Irie et al., 2015), or combination with TNAP in mice (Tang et al., 2015). In addition, human and mouse PGCs share several key genes that regulate germ cell fate, including BLIMP1 (PRDM1), SSEA1, OCT4, TFAP2C, DAZL, and DDX4 (Canovas et al., 2017). Expression of DDX4 (VASA) has been reported in several mammalian PGCs including human (Castrillon et al., 2000), rhesus macaques (Hermann et al., 2007), and mice (Rebourcet et al., 2016). DDX4/Ddx4 is a member of the DEAD-box family of protein and germline-specific RNA helicase which plays an important role in germ cell meiosis (Medrano et al., 2012) and is expressed in migratory PGCs in the region of gonadal ridge (Castrillon et al., 2000). Ddx4 has been demonstrated in mouse PSC-derived PGCLCs to retain the capacity of involvement in both spermatogenesis (Hayashi et al., 2011) and oogenesis (Ishikura et al., 2016). DDX4 has been reported to express a C-terminal domain (extracellularly; DDX4^ec^), with the N-terminal domain is located intracellularly (intracellularly; DDX4). Previous reports show that by DDX4 C-terminal antibody-based fluorescence-activated cell sorting (FACS), potential germline stem cells can be isolated from the human ovary (Zou et al., 2009) and show a functional ability to form oocyte-like structures (Clarkson et al., 2018; White et al., 2012). The transcriptome and proteome of human DDX4^ec^ PGCLCs derived from hPSCs have not yet been systematically analyzed, with the function of these cells remaining largely unknown. The role and function of DDX4^ec^ PGCLCs derived from differentiated human ESCs and iPSCs also remain especially un-established. Considering the critical role of DDX4 in the differentiation of human PSCs in germline lineage, it is important to analyze the role of DDX4^ec^ PGCLCs in germline development in the field of human PSC differentiation to germ cells in more details. Directed induction of PSCs to germ cells *in vitro* and *in vivo* evidently needs the appropriate and timely use of specific growth factors and/or microenvironment.

Previous studies show that activin A and basic fibroblast growth factor (bFGF) play important roles in the differentiation of mouse PGCLCs (mPGCLCs) from ESCs-derived EpiLCs, leading to a decrease in the expression of pluripotency markers and an increase in the expression of specific germ cell markers (Zhou et al., 2016). In addition, it has been demonstrated that activin A enhances the efficiency of human primordial follicle in oocyte development *in vitro* (Telfer et al., 2008). Retinoic acid (RA) signaling is essential during meiotic induction of PGCs. In a male mouse model, it was shown that the CYP26B1 regulates endogenous RA, which is induced by fetal-stage gonadal somatic cells to coordinate male germ cells into meiosis (MacLean et al., 2007; Zhou et al., 2016). In addition, several meiotic and self-renewal genes were also explicitly expressed in human female fetal germ cells in response to RA pathway signaling (Li et al., 2017; Zhou et al., 2016).

Previous studies also demonstrate that the three morphogens (activin A, BMPs and RA) regulate the expression of several germline genes and the initiation of meiosis in PGCs derived from ESCs (Koubova et al., 2014; Zhou et al., 2016). We made use of these morphogens for the initial induction of germ cells in this study. In addition, for further germ cell maturation and migration, a physiological niche microenvironment is usually needed. In terms of physiology, the movement of germ cells is important in determining germline developmental processes, regeneration, and cell migration. The expression of keratin (KRT) proteins such as KRT 8 and 18 can support the necessary shape changes and provide the stability needed for cell translocation (Seetharaman and Etienne-Manneville, 2020). Specifically for *in vivo* female germ cell growth, a stage of ovarian follicle formation is likely essential, in which the oocyte can be found to be surrounded by granulosa cells (GCs). Currently human GCs can be relatively easy to harvest during oocyte retrieval in an *in vitro* fertilization program, provided that IRB approval and patient informed consent are obtained. Previously we reported that human GCs can be successfully derived from hPSCs (Lan et al., 2013). These critical ovarian somatic cells thus can be used for the maturation of the DDX4^ec^ PGCLCs derived in this study.

Here, we tentatively named these DDX4^ec^-sorted hPSC-derived germ cells as DDX4^ec^ PGCLCs. We have conducted a comparative analysis of DDX4^ec^ PGCLCs derived from hESCs and hiPSCs and investigated the developmental potential of these cells in a xenograft animal model.

## Results

### Derivation of human PGCs from pluripotent stem cells

**Figure 1:**
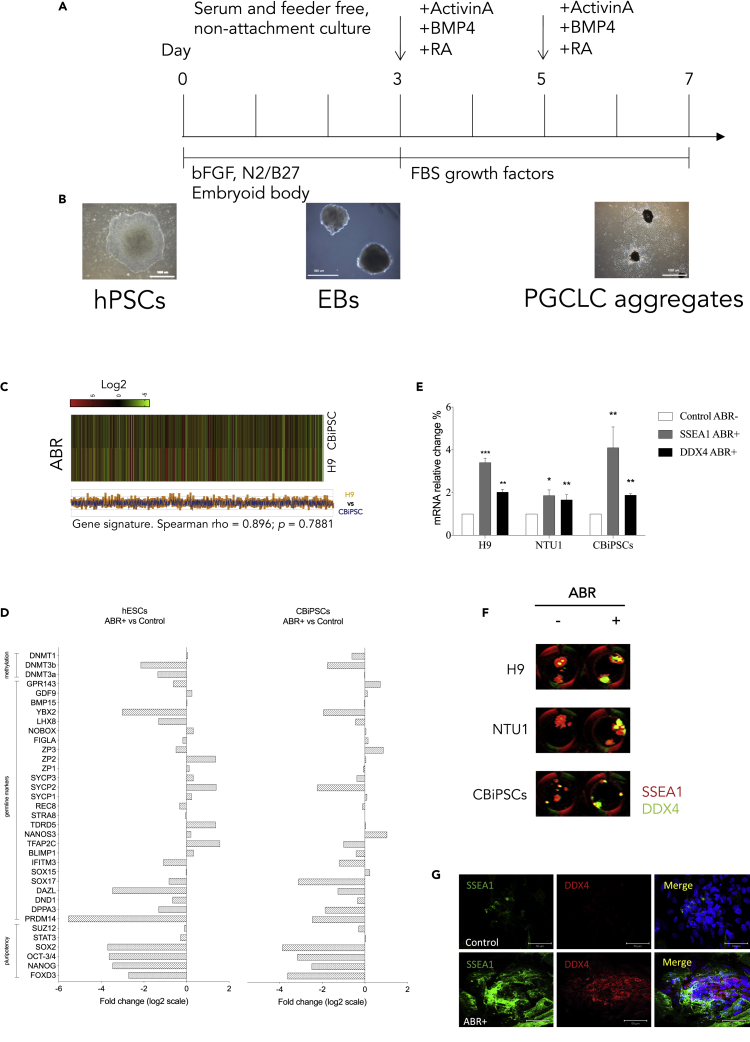
Differentiation of human pluripotent stem cells (PSCs) into human primordial germ cell-like cells (hPGCLCs) (A) Timeline and culture strategy of *in vitro* hPGCLC differentiation. (B) The morphology of human embryonic stem cells (hESCs), embryoid bodies (EBs) and hPGCLC aggregates induced by ABR (activin A, BMP4 and retinoic acid) treatment. (C) The comparison of transcriptional profiles of hPGCLC aggregates by ABR treatment between H9 hESCs and human cord blood cell-derived induced pluripotent stem cells (CBiPSCs). (D) The expression of germline makers in hPGCLC aggregates derived from H9 hESCs and CBiPSCs. (E and F) (E) The expression levels of DDX4 and SSEA1 mRNA and (F) protein under ABR treatment. (G) Immunofluorescent staining of DDX4 and SSEA1 in hPGCLC aggregates. Mann-Whitney *U* test was used for statistical analysis.Bar graph represents mean ± SEM from at least three independent experiments. ∗p < 0.05, ∗∗p < 0.01, ∗∗∗p < 0.001.

A schematic diagram of the differentiation strategy for human PGCLCs (hPGCLCs) formation is illustrated in Figure 1A. To generate hPGCLCs from hESCs and cord blood cell-derived iPSCs (CBiPSCs; a human iPSC line), we modified and improved the strategy described for mouse PGCLCs (mPGCLCs) (Oliveros-Etter et al., 2015) and hPGCLC specification in previous reports (von Meyenn et al., 2016; West et al., 2009). The hPSCs (Figure 1B) were cultured in a feeder-free and serum-free medium to generate hanging-drop embryoid bodies (EBs) by approximately 300 cells per drop maintained in bFGF/N2B27 medium (West et al., 2009). EBs formed after culturing for 3 days (Figure 1B). For PGCLC enrichment, EBs were transferred to DMEM-based medium supplemented with human activin A, BMP4, retinoic acid (ABR) and 15% fetal bovine serum (FBS). On day 5, the medium was re-supplemented with fresh ABR. On day 7, the differentiated cells were collected and investigated to assess the transcriptional signatures and mRNA and protein expression of DDX4 and SSEA1 (an early PGC marker). To characterize the PGCLC aggregates, we initially carried out an RNA-array to investigate the differences of the ABR-treated differentiated cells between hESCs and CBiPSCs (Figure 1C). The results of 2-fold change (FC > 2) ratios and the screened gene probe IDs for the comparison were submitted to identify the network connections. Gene Ontology (GO) analysis of the 3172 differentially expressed gene clusters revealed and identified the significant over-representation of 499/1413 signaling pathways (File S1). Our data showed that transcriptional signatures were very similar between the differentiated cells originating from hESCs and CBiPSCs by ABR treatment (Figure 1C, Spearman rho = 0.896 and p = 0.7881), including sharing the enrichment in several pathways of GO terms, such as biological process, cellular process, and multicellular organismal process (File S1). The differentiated cells of both origins lost some of the pluripotency markers and showed a decrease in methylation levels (Figure 1D), suggesting epigenetic reprogramming during *in vitro* PGCLC differentiation after ABR treatment. However, the differentiated cells derived from hESCs expressed higher levels of SYCP1, SYCP2, and SYCP3 than the cells derived from CBiPSCs, suggesting that the differentiated cells from hESCs might represent stronger potential for the initiation of meiosis than those of hiPSCs (Figure 1D). We next demonstrated that the mRNA (Figure 1E) and protein expression levels by immunofluorescence (Figure 1F) of DDX4 and SSEA1 significantly increased by using ABR treatment in PGCLC aggregates. Interestingly, we found that most of the DDX4-positive cells were not co-localized with SSEA1-positive cells (Figure 1G and see Figure S1A), suggesting the distinctive expressing pattern and timing of DDX4 in hPGCLCs.

### Characterization of DDX4^ec^ PGC-like cells

**Figure 2:**
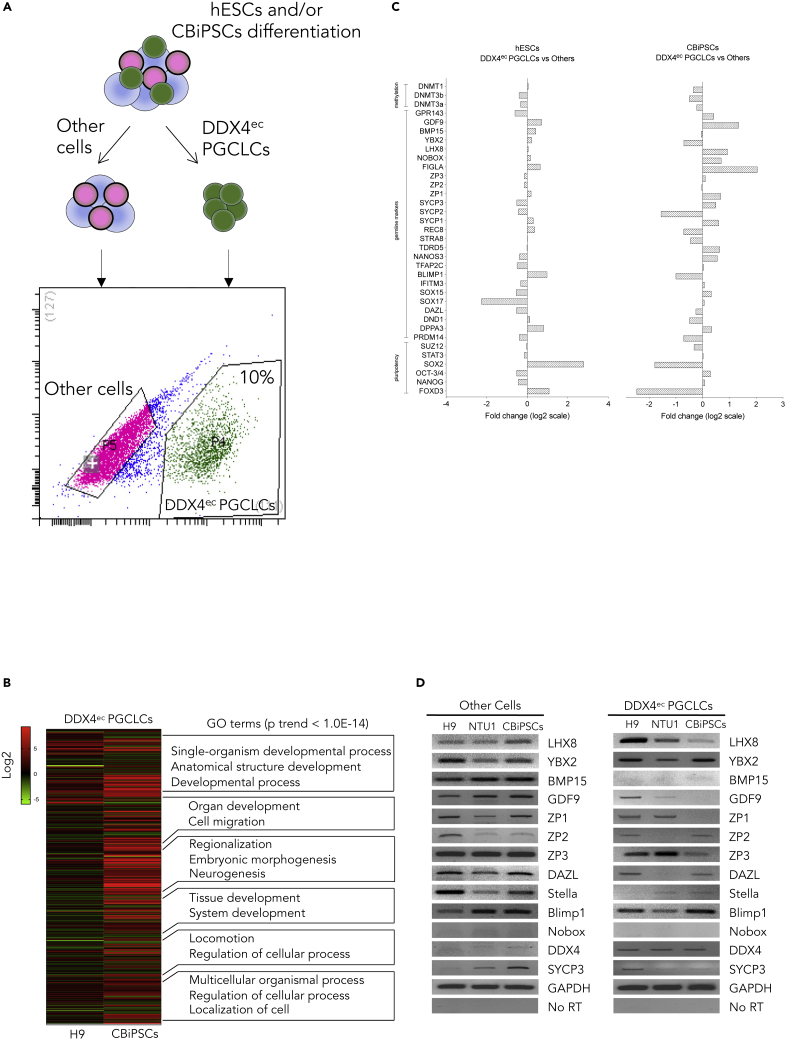
The transcriptional identity of human DDX4^ec^ primordial germ cell-like cells (PGCLCs) (A) Fluorescence-activated cell sorting (FACS) of DDX4 ^ec^ PGCLCs of day 7 hPGCLC aggregates. (B) The enriched GO term comparisons in DDX4^ec^ PGCLCs derived from H9 hESCs and CBiPSCs. (C) The expression of germline makers in DDX4^ec^ PGCLCs derived from hESCs and CBiPSCs. (D) Representative analysis of germline markers in FACS-sorted differentiated cells and DDX4^ec^ PGCLCs.

First, to demonstrate the ability of purifying DDX4^ec^ PGCLCs using a C-terminal extracellular epitope, we performed the double immunofluorescence analysis for DDX4^ec^ and E-cadherin in day 7 differentiated cells. Our results demonstrated that transmembrane protein E-cadherin confirmed the DDX4^ec^ PGCLC localization (Figure S1B). To deeply understand the role of DDX4^ec^ PGCLCs, we performed FACS to sort the DDX4^ec^ PGCLCs from the differentiated stem cells (Figure 2A). DDX4^ec^ PGCLCs can be isolated from approximately 10% day 7 differentiated cells after ABR treatment (Figure 2A). No significantly different percentages for DDX4^ec^ PGCLCs were observed between the differentiated cells originating in hESCs and CBiPSCs (Figure S2). To further characterize the DDX4^ec^ PGCLCs, we initially carried out an RNA-array to compare DDX4^ec^ PGCLCs derived from hESCs and CBiPSCs. We found that the transcriptional signatures of DDX4^ec^ PGCLC were differentially expressed between hESCs and CBiPSCs (Figure 2B). A heatmap of the 476 differentially expressed genes of pairwise comparisons and the GO analysis of the 311 differentially expressed gene clusters revealed that DDX4^ec^ PGCLCs derived from hESCs and CBiPSCs shared some enrichment in regard to single-organism developmental process, anatomical structure development, organ development, and cell migration (Figure 2B). Specifically, DDX4^ec^ PGCLCs derived from hESCs were enriched in GO terms in relation to regionalization and embryonic morphogenesis. In contrast, DDX4^ec^ PGCLCs derived from CBiPSCs were enriched during neurogenesis, including SPRY3, SEMA3A and SEMA4F (Figure 2B and File S2). Significantly, RNA-array data indicated that DDX4^ec^ PGCLCs derived from both hESCs and CBiPSCs expressed several PGC, folliculogenesis and postnatal oocyte-specific markers (Figure 2C). We next screened the mRNA expression levels in germline molecules. The expression levels of NOBOX and DDX4 were weak in the other differentiated cells, whereas LHX8, YBX2, BMP15, GDF9, ZP1, ZP2, ZP3, DAZL, Stella, Blimp1, and SYCP3 were expressed at higher levels in DDX4^ec^ PGCLCs (Figure 2D). No expression of BMP15 and NOBOX were observed in DDX4^ec^ PGCLCs and ZP1 was not expressed in DDX4^ec^ PGCLCs derived from CBiPSCs (Figure 2D).

### DDX4 PGCLCs are participating ovarian follicle-like structure formation

**Figure 3:**
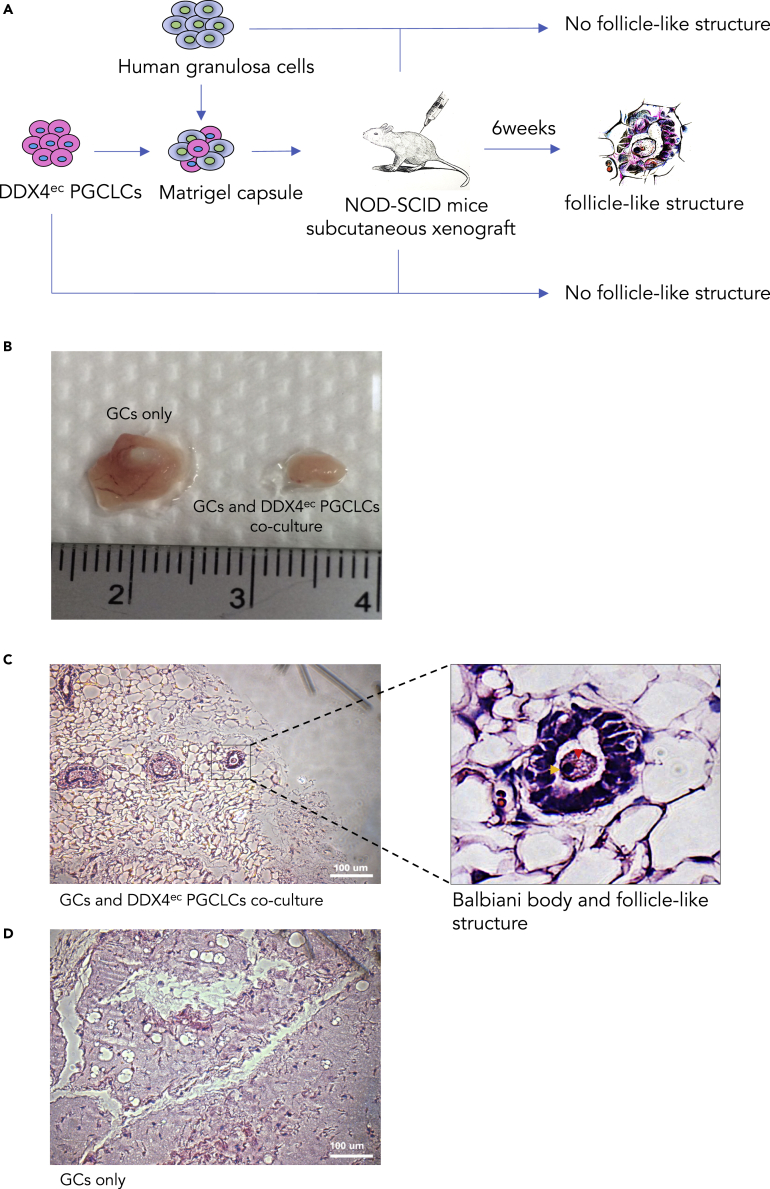
Human pluripotent stem cells (PSC) - derived human primordial germ cell-like cells (hPGCLCs) develop into ovarian follicle-like structures with a granulosa cell microenvironment in a NOD-SCID mouse model (A) Schematic timelines and the procedures for the DDX4^ec^ PGCLCs derived from hPSCs to the harvesting of transplants in the xenograft mouse model. (B) The transplants of human granulosa cells (GCs) with or without the presence of DDX4^ec^ PGCLCs. (C and D) (C) Ovarian follicle-like structures were obtained from the transplants of mixtures of GCs and DDX4^ec^ PGCLCs and (D) GCs only. The red arrow shows germinal vesicle-like staining and the orange arrow shows that the balbiani body is present in the follicle-like structures. The other dotted line areas show other follicle-like structures.

Physiologically, at the third to fifth week of gestation, hPGCs start to migrate from the yolk sac wall through the hindgut to the developing genital ridge. In order to further explore whether the DDX4^ec^ PGCLCs obtained in our culture were able to form ovarian follicle-like structures, subcutaneous xenografting was performed in NOD-SCID mice. After collecting DDX4^ec^ PGCLCs by FACS, the cells were mixed with human GCs, encapsulated by matrigel as an extracellular matrix (ECM) and transplanted subcutaneously in NOD-SCID mice and observed for 6 weeks (Figures 3A and S3). The tissues were then removed from mice (Figure 3B) after 6 weeks and examined. We observed that ovarian follicle-like structures appeared in the transplants both in the cells derived from hESCs and hiPSCs (Figure 3C (H9 hESCs) and Figures S4A and S4B (CBiPSCs)). Balbiani body (orange arrow) and germinal vesicle-like staining in the middle of the follicle-like structures (red arrow) accompanied with the surrounding cells suggested that these follicle-like structures were likely at the stage of primary follicles (Figure 3C). No follicle-like structures were observed in the GCs-only (Figure 3D) transplants. To further confirm the follicle-like structures to contained early stage germ cells and surrounding somatic cells, we stained the cells with DDX4 and AMHR2 (a GC marker). Immunostainings showed that these follicle-like structures expressed DDX4 in the center (presumably the oocyte) and AMHR2 at the peripheral locations, likely the surrounding GCs (Figure S4C). We also demonstrated AMHR2 but not DDX4 expression in the control GC transplants (Figure S4D), and no follicle-like structures were observed in GC control (Figure S4D) and DDX4^ec^ PGCLC control only (Figure S4E). Our data herein provide strong evidence that DDX4^ec^ PGCLCs were capable of developing into early ovarian follicle-like structures, when cultivated with human GCs. Taken together, after *in vitro* treatment with an appropriate cocktail of growth factors (activin A, BMP4 and retinoic acid) combined with a suitable *in vivo* environment with GC co-culture, human PSCs carry the potential to develop toward germ cell lineage, up to the stage of early ovarian follicles.

### Keratin 8 is predominantly expressed in DDX4ec PGCLCs

**Figure 4:**
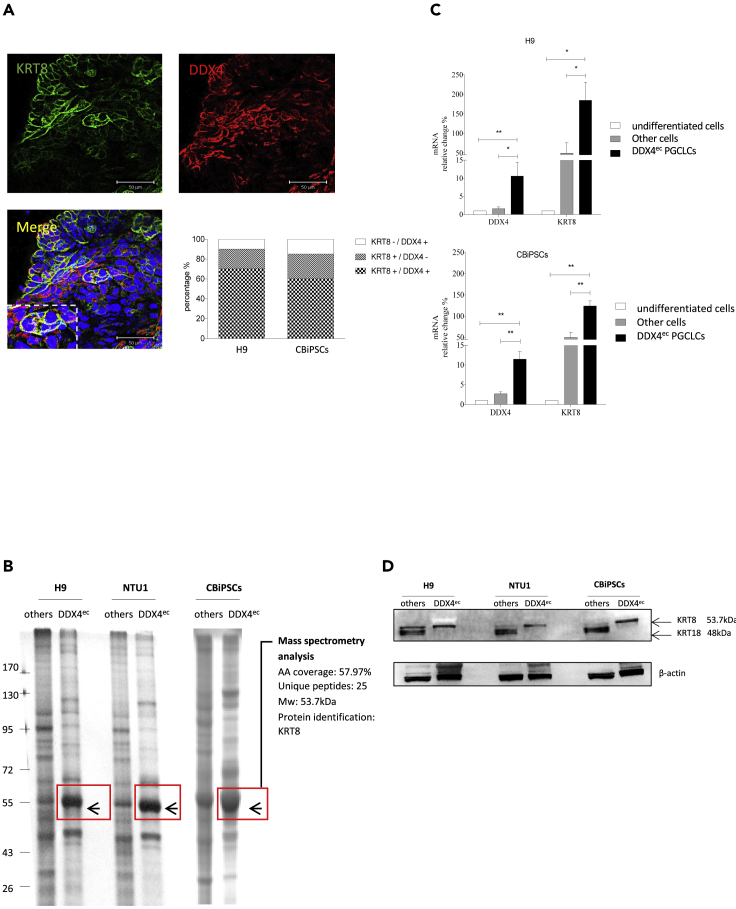
The proteomic identity of human DDX4^ec^ primordial germ cell-like cells (PGCLCs) (A) Immunofluorescent staining of DDX4 and KRT8 and the quantification of DDX4-expressing (DDX4^+^) and KRT8-expressing (KRT8^+^) cells at day 7 hPGCLCs. (B) Mass spectrometry analysis of the protein extracts in amino acids (AA) of DDX4^ec^ PGCLCs and the other differentiated cells derived from H9 hESCs and CBiPSCs. (C and D) (C) mRNA levels of DDX4 and KRT8 and (D) immunoblotting analysis of KRT8 in undifferentiated stem cells, DDX4^ec^ PGCLCs and the other differentiated cells derived from H9 hESCs and CBiPSCs. Mann-Whitney *U* test was used for statistical analysis. Bar graph represents mean ± SEM from at least three independent experiments. ∗p < 0.05, ∗∗p < 0.01. Mw: molecular weight.

From the RNA array analysis, we surprisingly found that KRT family member Keratin 8 (KRT8) was highly expressed in DDX4^ec^ PGCLCs compared with the other differentiated cells (File S2). To confirm the results, we stained the cells with DDX4 and KRT8 in 7 day PGCLC aggregates from hESCs and CBiPSCs. Here, we demonstrated that approximately over 50% of KRT8 positive cells were localized to the cell membranes of DDX4^ec^ PGCLCs (Figure 4A). We then conducted a comprehensive proteome analysis of sorted DDX4^ec^ PGCLCs from hESCs and CBiPSCs. The most predominant band in DDX4^ec^ PGCLCs derived from hESCs and CBiPSCs compared with the other differentiated cells were selected and analyzed by mass spectrometry. We found that KRT8 was the top high-level protein species in the proteins (Figure 4B). We next confirmed that the mRNA expression levels of DDX4 and KRT8 in DDX4^ec^ PGCLCs derived from hESCs and CBiPSCs were significantly higher than the undifferentiated hESCs, the undifferentiated CBiPSCs and the other differentiated cells (Figure 4C). We further demonstrated that KRT8 was predominantly expressed in DDX4^ec^ PGCLCs both from hESCs and CBiPSCs, and that KRT8 (lower level) and KRT18 (higher level) were also detectable in the other differentiated cells by using immunoblotting (Figure 4D). Our data suggest that KRT8 might be an important marker in PGC developmental progression. Since keratin family is involved in cytoskeletal organization and KRT8 is highly associated with cell migration, cell adhesion, and metastasis in some tumors (Fang et al., 2017), we assume that KRT8 might play an important role in germ cell migration toward the gonadal ridge.

### Keratin 8 is essential in DDX4 migratory PGCs

**Figure 5:**
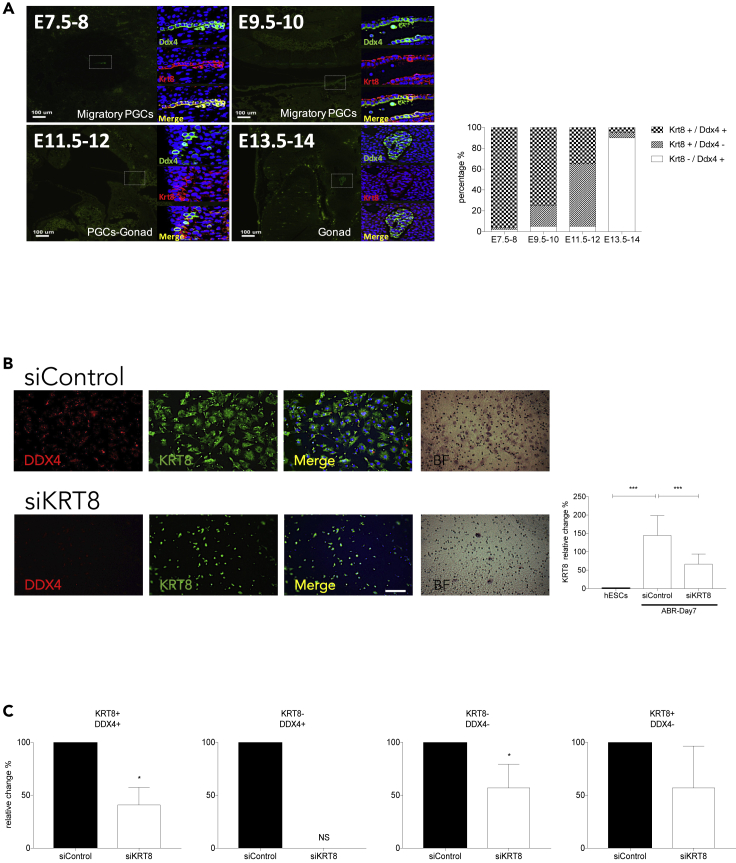
KRT8/Krt8 is expressed and essential in mouse primordial germ cells (PGCs) and human primordial germ cell-like cells (hPGCLCs) migration (A) Immunohistochemistry staining of Ddx4 and Krt8 and the quantification of Ddx4-expressing (Ddx4^+^) and Krt8-expressing (Krt8^+^) cells at different days of mouse embryos. (B) KRT8 knockdown (KRT8-KD) cells were generated using siRNA transfection and KRT8 mRNA levels were analyzed by qRT-PCR in hPGCLCs. Representative immunofluorescent images of filters stained with DDX4 and KRT8 in a trans-well cell migration assay. KRT8-KD or siControl cells were plated in the upper chamber and the migration was stimulated by the addition of 10% FBS. The migratory cells were also stained with hematoxylin-eosin (right panels). Scale bar, 100um. (C) The migratory cells were quantified by counting the DDX4^+^ and KRT8^+^ cells in several random fields. Mann-Whitney *U* test was used for statistical analysis. Bar graph represents mean ± SEM from at least three independent experiments. ∗p < 0.05, ∗∗∗p < 0.001.

In order to confirm our hypothesis, we examined the expression of Krt8 and Ddx4 in mouse PGCs from E8-E14 (E: embryonic day) using immunofluorescence. We identified Krt8 on the surfaces of almost all Ddx4^+^ cells in migratory PGCs from E7.5 to E10 (Figure 5A). However, from E11.5 and E13.5 onward in migratory PGCs, Krt8, and Ddx4 proteins became uncoupled, with 30% (E11.5) and less than 10% (E13.5) of Krt8^+^ cells co-expressed with Ddx4^+^ (Figure 5A). Therefore, by demonstrating Krt8 and Ddx4 expression in this mouse model, we propose a common PGC migratory stage in mice from E8 to E14, in which Krt8 might be playing an important role in the regulation of PGC migration. To further reinforce our hypothesis in human PGCs, we assessed the chemotactic activity of KRT8-knockdown (KRT8-KD) and control differentiated cells using trans-well migration assays. The KRT8 mRNA levels were low in undifferentiated hESCs and were increased on day 7 in the ABR-treated differentiated cells (Figure 5B). As expected, the KRT8 mRNA levels were significantly reduced in the siKRT8-KD cells (KRT8-KD by siRNA) when compared with the control-KD cells (Figure 5B). Subsequently, we observed that the migration of KRT8-KD DDX4^+^ PGCLCs was significantly reduced in comparison to the control cells in response to 10% FBS gradients across gelatin-coated membranes in the trans-well migration assay (Figure 5B). By examining the cells which successfully migrated through the membranes, we found that the number of migratory KRT8^+^/DDX4^+^ cells (cells-expressing both KRT8 and DDX4) were reduced by approximately 50% in KRT8-KD DDX4^+^ PGCLCs, when compared with the control cells (Figure 5C). No KRT8^-^/DDX4^+^ cells (cells-expressing DDX4 but not KRT8) were observed in the migratory cells, suggesting the critical role of KRT8 expression in DDX4^+^ cell migration (Figure 5C). In addition, the number of KRT8^-^/DDX4^-^ cells in the migratory cell fraction of KRT8-KD group was also reduced in comparison to the migration of control cells (Figure 5C). Our results therefore suggest that KRT8/Krt8 is required for hPGC and mouse PGC migration.

## Discussion

Using PSCs to generate functional mature germ cells has long been considered, with the concept confirmed in the reconstitution of oogenesis in mouse models (Hayashi et al., 2012; Hikabe et al., 2016; Yamashiro et al., 2020). Recent studies have also successfully established early human PGCs and human oogonia, an immediate embryonic precursor for human oocytes from PSCs, but mature gametes have not yet been consistently derived from hPSCs, except recent reports about the feasibility of generating human oocytes in culture (Yamashiro et al., 2018, 2020). These results suggest although human PGCs likely have a unique gene profile, the developmental mechanisms of human PGCs into germ cells may be substantially similar to the other species, particularly in epigenetic reprogramming. Therefore, using the age-matched endogenous ovary tissue that the human stem cell-derived hPGCLCs might gain developmental competence (Irie et al., 2015; Kobayashi et al., 2017; Tang et al., 2015). DDX4 encodes an ATP-dependent RNA helicase and it is known that DDX4 is a germline-specific molecule in various organisms. In mammals, DDX4 is found to be expressed specifically in PGCs and mature germ cells in most phyla studies, suggesting that DDX4 plays a crucial role in germline development and maintenance. DDX4 has been reported to have a C-terminal domain which is expressed extracellularly (Zou et al., 2009). To explore the ill-defined role of DDX4^ec^ PGCLCs, we here report the use of strategies in terms of a cocktail of several morphogens to drive the development of DDX4^ec^ PGCLCs from human PSCs. Subsequently, a further appropriate *in vivo* niche environment created by co-cultivating with human GCs enhanced the formation of early ovarian follicle-like structures. We also observed the potentially critical role of KRT8 in the migration of DDX4^ec^ PGCLCs to the genital ridge.

Resetting of the epigenome in hPGCs is critical for germline lineage development. Our data show that the pluripotency genes were downregulated by activin A, BMP4 and retinoic acid treatment. Furthermore, DDX4^ec^ PGCLCs derived from hESCs and hiPSCs exhibited differential transcriptome signatures, though both cell types still share similar germline differentiation potential. For example, the DDX4^ec^ PGCLCs derived from hPSCs (both hESCs and hiPSCs) exhibited several germline markers including DPPA3, BLIMP1, and meiotic makers including REC8, SYCP1, and folliculogenesis markers including ZP1, 2, 3, GDF9 and FIGLA. The expression of DNMT1, DNMT3a and DNMT3b decreased in DDX4^ec^ PGCLCs, indicating the chromatin reorganization and comprehensive DNA hypomethylation. For this latter issue, a further in-depth study is necessary for confirmation, after the efficiency of cell differentiation can be further enhanced. We thus have good reasons to consider that these DDX4^ec^ PGCLCs have likely exited the pluripotent state and entered meiosis, a critical step in germline development. Taken together, our data suggest that DDX4^ec^ PGCLCs obtained in this study are similar to early native PGCs but also express some mid-late PGC markers, implying that these cells retain the potential to develop into more advanced-stage oocytes such as oocytes in the ovarian primary or secondary follicles. We thus examined if DDX4^ec^ PGCLCs could be utilized to induce human folliculogenesis *in vitro*. GCs are somatic cells surrounding the oocytes in the mammalian ovarian follicles. It is known that GCs interact intimately with oocytes and can secret growth factors and sex steroids to create a niche environment for the development and maturation of oocytes by paracrine and autocrine effects (Wigglesworth et al., 2013; Zhang et al., 2010). GCs, therefore are essential for folliculogenesis and are indispensable for ovarian follicle formation and growth at various stages. In this report, we successfully demonstrate the possibility of the growth of PSC-derived DDX4^ec^ PGCLCs toward the ovarian primary follicle stage, though efficiency remains low. This observation is important since the step-by-step improvement of each step of ovarian follicle growth and maturation from PSC-derived PGCLCs will hopefully help produce a final harvest of mature follicles or oocytes that provides an *in vitro* model of research for gamete biology.

Mammalian germ cells are specified at the border between embryonic and extraembryonic tissues (Richardson and Lehmann, 2010). Subsequently, germ cells must migrate through and along various somatic tissues to reach and aggregate the niche of the gonad. Previous studies show that mouse germ cells depart from the hindgut by the bFGF/Wnt signaling pathway and then undergo a trans-epithelial migration (Chawengsaksophak et al., 2012; Takeuchi et al., 2005). Dynamic adhesion to the ECM is observed during these processes in germ cells of some species. For instance, isolated migratory mouse germ cells during exit from the hindgut display more E-cadherin expression than germ cells isolated at the end of migration (García-Castro et al., 1997). The keratin family is a major component of the intermediate filament which connects to ECM and regulates cell adhesion and migration. Interestingly in this study, the data show that human PSC-derived DDX4^ec^ PGCLCs express a significant increase in KRT family members, particularly KRT8, an observation further supported by the finding that migratory mouse PGCs also express high levels of Krt8 during the migration period when compared with the germ cells in the gonad. We further confirmed the importance of KRT8 expression in human PGC migration by demonstrating that KRT8 deficiency led to a profound impairment in human PGC migration. Taken together, these human and mouse studies strongly support the notion that keratin family member, especially KRT8, very likely play a critical role in mammalian germ cell migration to the genital ridge.

In conclusion, we have identified a critical role of KRT8 in the chemotactic migration of human PGCLCs derived from PSCs, which may direct our further study into the currently un-established mechanisms of human germ cell migration. Our data also suggest that when an appropriate niche environment is provided, DDX4^ec^ PGCLCs can exit a pluripotency state and progress into mid-stage germ cells, showing evidence of meiosis and the potential of ovarian folliculogenesis. We previously showed that human GCs can be successfully derived from hESCs and hiPSCs (Lan et al., 2013). Thus by using the human PGCLCs derived in this study and the previously reported stem cell-derived GCs (Lan et al., 2013), both originating from autologous hiPSCs, we can explore the mechanisms of germ cell development in a fully autologous system and hopefully design applicable strategies for some important causes of infertility such as primary ovarian insufficiency or aged-related reduced oocyte quality and quantity. Finally, it is hoped that our findings provide new insights into unique human germline development through an artificial cell model by way of *in vitro* and *in vivo* human PSCs differentiation.

### Limitations of the study

The primary follicle-like structure in our study is developed from human stem cells derived-DDX4ec PGCLCs capsuled with human GCs and transplanted subcutaneously in NOD-SCID mice. The primary follicle-like structure here is an early stage and the exogenous GCs might present in any place including developmental follicle-like structure. It is challenging to get similar or parallel human primary follicle samples as a positive control demonstrating our results because it will be from the human fetal ovary. Future research is needed to identify the possible parallel human primary follicles.

### Resource availability

#### Lead contact

Further information and requests for resources and reagents should be directed to and will be fulfilled by the lead contact, DannyCw Yu (dcwyu@email.nchu.edu.tw).

#### Materials availability

The materials in this study will be available on the requests with a completed materials transfer agreement.

#### Data and code availability

This study did not generate code and the RNA array and mass spectrometric data can be downloaded from the supplemental file.

## Methods

All methods can be found in the accompanying Transparent methods supplemental file.

## Supplemental information

Document S1. Transparent methods, Figures S1–S4, and Table S1

File S1. Gene clusters, related to figure 1

File S2. PGCLCs enriched genes, related to figure 2
