# Supplementary material for: Human pluripotent stem cell-derived DDX4 and KRT-8 positive cells participate in ovarian follicle-like structure formation
Source: iScience. 2020 Dec 29;24(1):102003. doi: 10.1016/j.isci.2020.102003 (PMC7811146; doi:10.1016/j.isci.2020.102003)
Supplement: Document S1. Transparent methods, Figures S1–S4, and Table S1 [file mmc1.pdf]

iScience, Volume *24*

Supplemental Information

Human pluripotent stem cell-derived DDX4 and KRT-8 positive cells participate in ovarian follicle-like structure formation

Danny C.W. Yu, Fang-Chun Wu, Chia-Eng Wu, Lu-Ping Chow, Hong-Nerng Ho, and Hsin-Fu Chen

Transparent Methods

Human Cells, PSC Culture and Differentiation

Human H9 ESCs (hESCs; karyotype 46, XX; WiCell, WI, USA) (Thomson et al., 1998) and NTU1 hESCs (karyotype 46, XX, derived in this lab) (Chen et al., 2007) were used in the present study. Culture protocols of hPSCs were modified and described from our previous reports (Chen et al., 2009a; Chen et al., 2009b; Chen et al., 2007; Robertson et al., 2007). The CBiPSCs (CB: cord blood) were generated using human cord blood-derived $CD34^+$ progenitors with seven episomally expressed factors (Cat# A18945, Life Technologies) (Burridge et al., 2011). The hPSCs (both hESCs and hiPSCs) were continuously maintained on mitomycin-C treated mouse embryonic fibroblasts using ReproCELL serum-free medium (ReproCELL, Tokyo, Japan) and passaged weekly manually using 30-gauge insulin needles as described from our previous protocols (Chen et al., 2009b; Chen et al., 2007). Differentiation of PGCs was performed according to the protocols (Irie et al., 2015; Vincent et al., 2013). Briefly, embryoid bodies (EBs) were generated by putting approximately 300 PSCs in each drop and maintained in bFGF/N2B27 medium (West et al., 2009). On day 5 and 7, EBs were transferred to DMEM-based medium supplemented with 20 ng/ml activin A, 100 ng/ml BMP4 and 0.1 uM RA (the ABR combination). Human granulosa cells (GCs) were obtained from ovarian follicular aspirates during oocyte retrieval in *in vitro* fertilization programs conducted in National Taiwan University Hospital. The use of human cells had been approved by the Institutional Review Board of Academia Sinica and the Ethical Committee of National Taiwan University Hospital. Informed consents were obtained from all subjects.

Animals and Xenograft

Different days of HaM/ICR mouse embryos were purchased from BioLASCO, Taipei, Taiwan and College of Medicine, National Taiwan University. Briefly, slides of the embryos were formalin-fixed, paraffin-embedded and sectioned. Immunodeficient NOD/SCID mice (BioLASCO, Taipei, Taiwan) were used in the xenograft study. 1 x 10^5 FACS-sorted $DDX4^{ec}$ PGCLCs were mixed with 1 x 10^6 human GCs and embedded in Matrigel (an extracellular matrix; ECM). The cell capsules were transplanted into the backs of mice *via* subcutaneous injections. After 6 weeks, mice were sacrificed by carbon dioxide and the newly formed tissues were washed with DPBS and fixed in 10% buffered formalin before being paraffin-embedded. All procedures had been approved by the Institutional Review Board of the Ethical Committee for Animal Study of National Taiwan University Hospital.

RNA extraction, cDNA Preparation and Quantitative RT-PCR (qPCR)

Total RNA was harvested by Trizol® reagent (Invitrogen) following the manufacturer's instruction. Reverse transcription was performed using the Maxima First Strand cDNA Synthesis Kit (Thermo Scientific, #L1642). The following qPCR procedure was initiated by 3 minutes at 95 degree, and followed by 40 cycles of 15 seconds at 95 degree, 30 seconds at 55 degree, and 30 seconds at 72 degree. The expression levels of the target genes were calibrated by the internal control GAPDH and the collected data were analyzed by ABI 7500 Fast Real-Time PCR system. Quantitative PCR (qPCR) reaction was performed on 1x EvaGreen reagent (Biotium, Fremont, CA, USA, catalog number 31014); 1 ul of diluted cDNA and 100 uM selected primers (IDT, San Jose, CA, USA, Table S1) were used at 2ul in 20ul of qPCR reaction. Quantification of all the samples by the software was calculated by the C_T and relative fold changes were calculated using the $2^{-\Delta\Delta CT}$ (Yu et al., 2016).

Immunocytochemistry, immunohistochemistry and Fluorescence Activated Cell Sorting

The anti-human SSEA1 (MAB2155, R&D system), DDX4 (ab13840, Abcam), KRT8 (sc-52324, Santa Cruz) and E-cadherin (AF748, R&D system) antibodies were used at the dilution of 1:200. The secondary antibodies were Alexa Fluor 594 goat anti-mouse IgG, donkey anti-goat IgG, Alexa Fluor 488 goat anti-mouse IgM and donkey anti-rabbit IgG (Abcam and Molecular Probes, Eugene, OR, USA). The presence of expression of SSEA1 and DDX4 in the cells and mouse tissues were measured with the NIS-elements BR2.30 software (Nikon, Tokyo, Japan) and Carl Zeiss LSM 780, and the numbers of immune-reactive cells in these areas were quantified in a blinded fashion. $DDX4^{ec}$ cells were isolated by FACS Aria II benchtop flow cytometer running DiVa 6 software. The unlabeled and secondary antibody only–labelled dissociated differentiated hPSC samples are used each time as controls to gate against background autofluorescence and to rule out non-specific binding of the secondary antibody. DDX4 antibody was used at 1:200 dilutions.

Mass Spectrometric Analysis

The protein samples from $DDX4^{ec}$ cells and $DDX4^{ec}$-negative cells were determined by SDS page electrophoresis and specific bands were extracted and analyzed by mass spectrometric analysis. Briefly, each sample was reduced with 5mM tris-carboxyethyl phosphine hydrochloride (TCEP) for 1h at 37°C, alkylated with 10 mM methylethanethiosulfonate (MMTS) for 20 min at room temperature (RT). Each dried fraction was reconstituted in 100 ul of 0.1% formic acid and 2% acetonitrile and then analyzed using a Q-Star Elite mass spectrometer (Applied Biosystems Company; MDS-Sciex), coupled to a prominence HPLC system (Shimadzu). The mass

spectrometer was set to perform data acquisition in the positive ion mode, with a selected mass range of 300 – 2,000 m/z. Peptides with +2 to +4 charge states were selected for MS/MS and the time of summation of MS/MS events was set to 2s.

Immunoblotting

Cells were washed with cold DPBS twice before being lysed in RIPA buffer (BioProducts, Ashland, MA, USA) supplemented with phosphatase inhibitor cocktail set II (brand, Calbiochem, San Diego, CA). Immunoblotting analysis was performed as described from our previous report (Yu et al., 2016). All primary antibody incubations were performed at 4°C overnight at the following dilutions: SSEA1 1:1000; DDX4 1:1000; KRT8 1:1000, and mouse monoclonal anti-actin (Sigma), 1:10,000.

Transcriptional Profiling of DDX4 cells by RNA Array

0.2 ug of total RNA was amplified by a Low Input Quick-Amp Labeling kit (Agilent Technologies, USA) and labeled with Cy3 (CyDye, Agilent Technologies, USA) during the in vitro transcription process. 0.6 ug of Cy3-labeled cRNA was fragmented to an average size of about 50-100 nucleotides by incubation with fragmentation buffer at 60°C for 30 minutes. The labeled cRNA was hybridized to Agilent SurePrint Microarray (Agilent Technologies, USA) at 65°C for 17 h. After washing and drying, microarrays are scanned with an Agilent microarray scanner (Agilent Technologies, USA) at 535 nm for Cy3. Raw signal data were normalized by quantile normalization for differential expressed genes discovering. For the functional assay, we used cluster-profile for enrichment test for gene ontology (GO) and pathway (KEGG).

RNA Interference

On-target KRT8 plus SMARTpool (SR302611) and control siRNA were purchased from OriGene (Rockville, MD, USA). The siRNA of KRT8 was 3 unique 27mer siRNA duplexes targeting Locus ID 3856. The siRNAs were transfected into the cells in 12-well plate using DharmaFECT1 transfection reagent according to manufacturer's instructions. 6h after transfection, the media was replaced by fresh media. After further 24h, the cells were collected for further analysis.

Migration Assays

Chemotaxis/directed migration assay was performed using polycarbonate filter wells (transwell, 8-lm pores; Coaster, Corning, NY) coated with 1% gelatin. Transwell migration of DDX4^{ec} PGCLCs was stimulated by 10% FBS to the culture medium in the lower well. After 24h, the upper surface of the insert was swabbed to remove non-migrating cells. The cells that had migrated to the lower surface were fixed and

recognized by HE stains (Dade Behring, Deerfield, IL). Cells migration was quantified by counting the number of cells in three random fields per insert.

Statistical Analysis

All results are presented as mean +/- standard error of the mean from a minimum of three independent experiments. Mann-Whitney U test was used to determine statistical significance and $p<0.05$ was considered significant.

Reference:

Burridge, P.W., Thompson, S., Millrod, M.A., Weinberg, S., Yuan, X., Peters, A., Mahairaki, V., Koliatsos, V.E., Tung, L., and Zambidis, E.T. (2011). A universal system for highly efficient cardiac differentiation of human induced pluripotent stem cells that eliminates interline variability. PLoS One *6*, e18293.

Chen, H.F., Chuang, C.Y., Shieh, Y.K., Chang, H.W., Ho, H.N., and Kuo, H.C. (2009a). Novel autogenic feeders derived from human embryonic stem cells (hESCs) support an undifferentiated status of hESCs in xeno-free culture conditions. Hum Reprod *24*, 1114-1125.

Chen, H.F., Kuo, H.C., Chen, W., Wu, F.C., Yang, Y.S., and Ho, H.N. (2009b). A reduced oxygen tension (5%) is not beneficial for maintaining human embryonic stem cells in the undifferentiated state with short splitting intervals. Hum Reprod *24*, 71-80.

Chen, H.F., Kuo, H.C., Chien, C.L., Shun, C.T., Yao, Y.L., Ip, P.L., Chuang, C.Y., Wang, C.C., Yang, Y.S., and Ho, H.N. (2007). Derivation, characterization and differentiation of human embryonic stem cells: comparing serum-containing versus serum-free media and evidence of germ cell differentiation. Hum Reprod *22*, 567-577.

Irie, N., Weinberger, L., Tang, W.W., Kobayashi, T., Viukov, S., Manor, Y.S., Dietmann, S., Hanna, J.H., and Surani, M.A. (2015). SOX17 is a critical specifier of human primordial germ cell fate. Cell *160*, 253-268.

Robertson, N.J., Brook, F.A., Gardner, R.L., Cobbold, S.P., Waldmann, H., and Fairchild, P.J. (2007). Embryonic stem cell-derived tissues are immunogenic but their inherent immune privilege promotes the induction of tolerance. Proc Natl Acad Sci U S A *104*, 20920-20925.

Thomson, J.A., Itskovitz-Eldor, J., Shapiro, S.S., Waknitz, M.A., Swiergiel, J.J., Marshall, V.S., and Jones, J.M. (1998). Embryonic stem cell lines derived from human blastocysts. Science *282*, 1145-1147.

Vincent, J.J., Huang, Y., Chen, P.Y., Feng, S., Calvopiña, J.H., Nee, K., Lee, S.A., Le, T., Yoon, A.J., Faull, K., *et al.* (2013). Stage-specific roles for tet1 and tet2 in DNA demethylation in primordial germ cells. Cell Stem Cell *12*, 470-478.

West, J.A., Viswanathan, S.R., Yabuuchi, A., Cunniff, K., Takeuchi, A., Park, I.H.,

Sero, J.E., Zhu, H., Perez-Atayde, A., Frazier, A.L., *et al.* (2009). A role for Lin28 in primordial germ-cell development and germ-cell malignancy. Nature *460*, 909-913.
Yu, C.W., Liang, X., Lipsky, S., Karaaslan, C., Kozakewich, H., Hotamisligil, G.S., Bischoff, J., and Cataltepe, S. (2016). Dual role of fatty acid-binding protein 5 on endothelial cell fate: a potential link between lipid metabolism and angiogenic responses. Angiogenesis *19*, 95-106.

Supplementary Figures

(A)

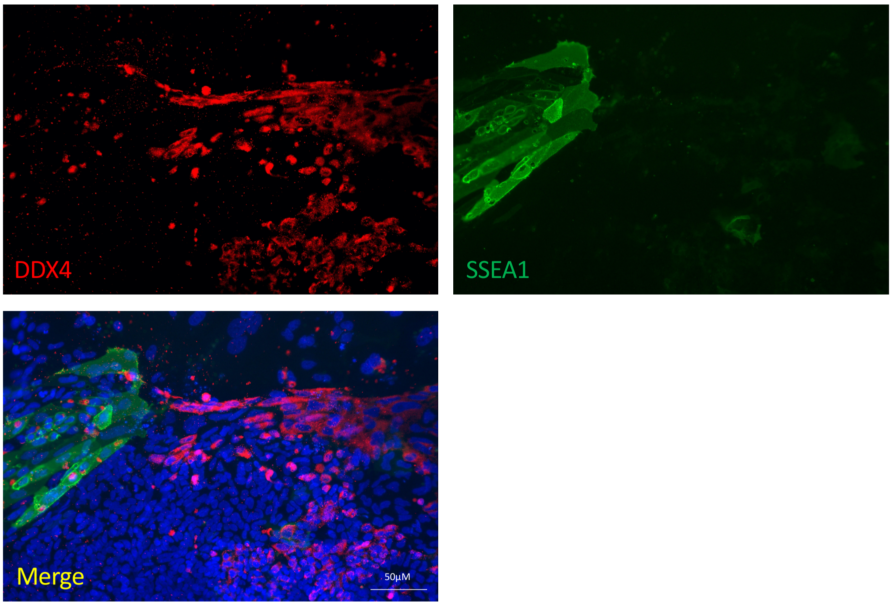

(B)

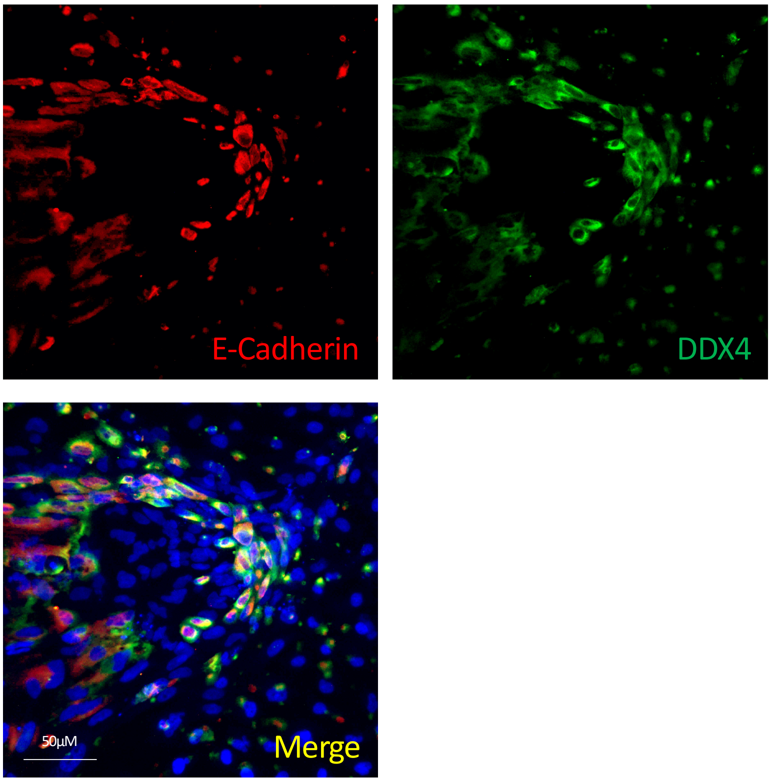

Figure S1. DDX4, SSEA1 and E-Cadherin expression in day 7 differentiated cells, Related to Figure 1.

(A) Double immunocytofluorescence for DDX4 (red) and SSEA1 (green) in hPGCLCs shows uniform expression of DDX4. (B) Transmembrane protein E-cadherin (red) is colocalized with $DDX4^{ec}$ (red) PGCLCs.

(A)

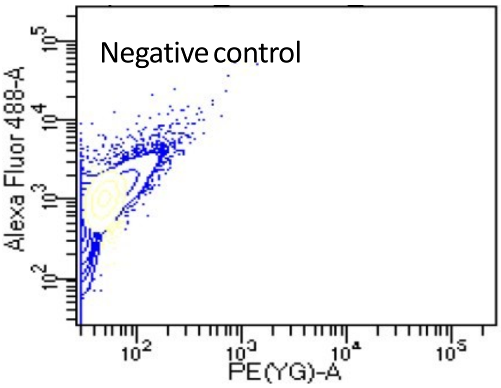

(B)

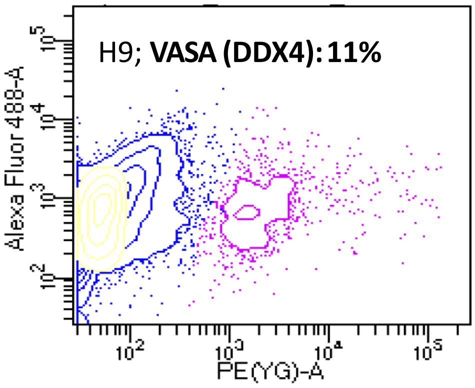

(C)

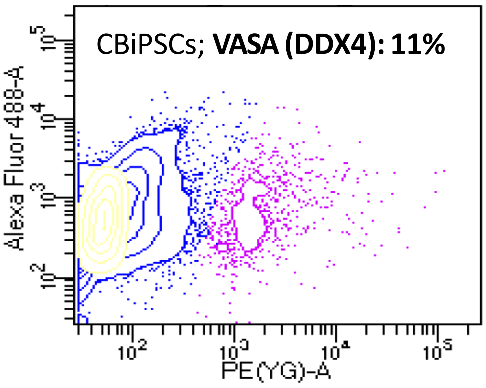

Figure S2. The DDX4^{ext} PGCLCs were derived from HPSCs and analyzed by flow cytometry, Related to Figure 2.

(A) The negative control showed no DDX4 positive cells. The percentage of DDX4^{ext} PGCLCs derived from (B) H9 (hESCs) (C) CBiPSCS showed no significant difference.

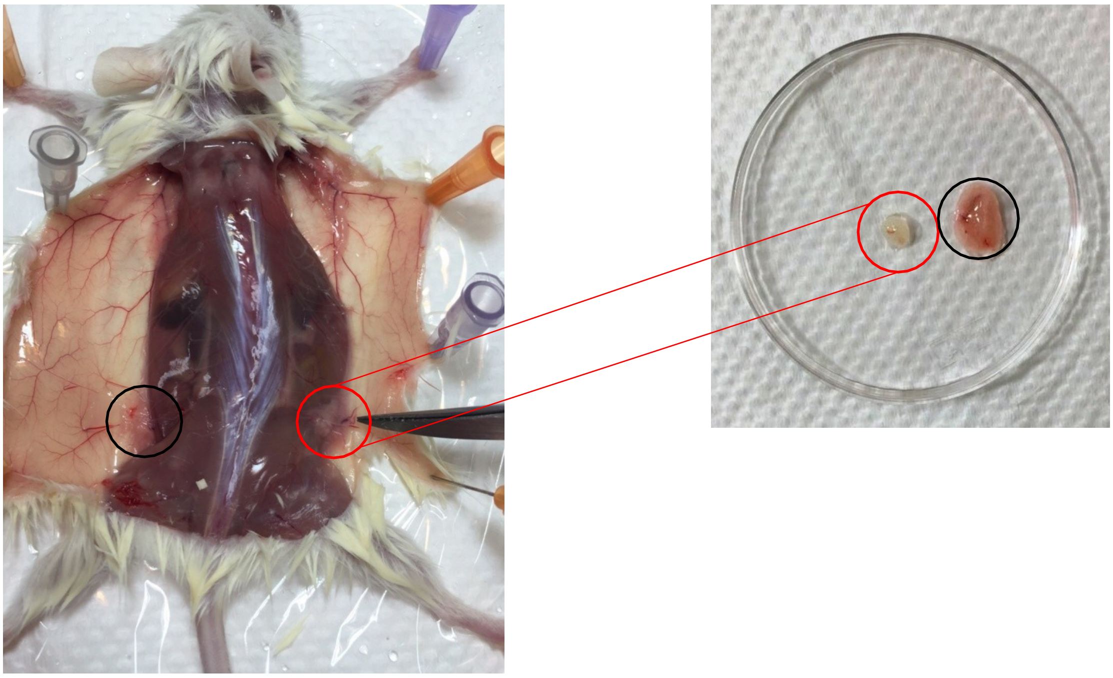

Figure S3. DDX4^{ext} PGCLCs and human granulosa cells transplanted into mouse after 6weeks, Related to Figure 3.

The red area was DDX4^{ext} PGCLCs mixed with human GCs and the black area was human GCs only.

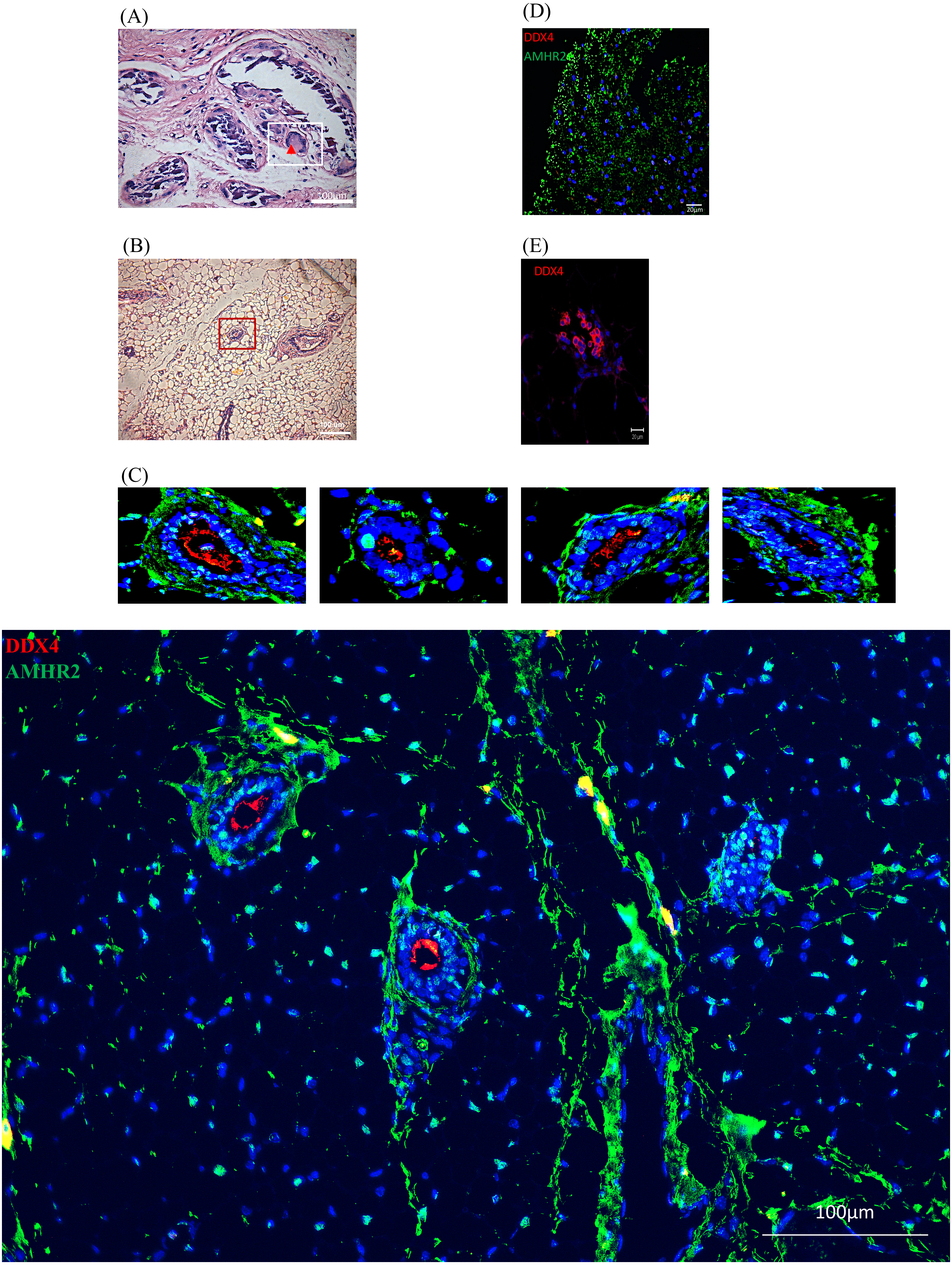

(A)
100µm
(B)
100 µm
(C)
(D)
DDX4
AMHR2
20µm
(E)
DDX4
20µm
DDX4
AMHR2
100µm

Figure S4. Human Follicle-like structures, Related to Figure 4, were developed from DDX4^{ec} PGCLCs derived from (A) H9 (hESCs), (B) CBiPSC (hiPSCs) in a xenograft model. Human Follicle-like cells were stained with DDX4 and AMHR2 in (C) DDX4^{ec} PGCLCs co-culture with GCs transplant and (D) GCs transplant only and (E) DDX4^{ec} PGCLCs only in a xenograft model. The arrow and dotted lines showed new formed follicle-like cells. Scare bars represent in the figures.

Supplementary Table:

Table S1. Primer list, Related to Figure 2.

	DAZL (5'→3')
F	TCGAACTGGTGTGTCCAAAGGCTA
R	TAGGATTCATCGTGGTTGTGGGCT
	YBX2 (5'→3')
F	ACCCTACCCAGTACCCTGCT
R	GCAAGAAAAGCAACCAGGAG
	SYCP3 (5'→3')
F	TATGGTGTCCTCCGGAAAAA
R	AACTCCAACTCCTTCCAGCA
	NOBOX (5'→3')
F	ATAAACGCCGAGAGATTGCCCAGA
R	AAGTCTGGTCAGAAGTCAGCAGCA
	LHX8 (5'→3')
F	CAAGCACAATTTGCTCAGGA
R	GGCACGTAGGCAGAATAAGC
	GDF9 (5'→3')
F	TCACCTCTACAACACTGTTCGGCT
R	AAGGTTGAAGGAGGCTGGTCACAT
	VASA (5'→3')
F	TTGTTGCTGTTGGACAAGTGGGTG
R	GCAACAAGAACTGGGCACTTTCCA
	KRT8 (5'→3')
F	ACCATGTCCATCAGGGTGAC
R	AGAAGCTCGAGGAGCTGATG
	GAPDH (5'→3')
F	ACAGTCAGCCGCATCTTCTT
R	GGCAACAATATCCACTTTACC
	OCT4 (5'→3')
F	GACAGGGGGAGGGGAGGAGCTAGG
R	CTTCCCTCCAACCAGTTGCCCCAAAC
	STELLA (5'→3')
F	GCGGAGTTCGTACGCATGA

R	CCATCCATTAGACACGCAGAAA
	BLIMP1 (5’→3’)
F	TGGAGAAACGGCCTTTCAAAT
R	CCTGGCATTCATGTGGCTTT
	ZP1 (5’→3’)
F	TGCTCCATCTCTGCTACCACTG
R	GTCTTGTGCCACATCCACAC
	ZP2 (5’→3’)
F	CTCAGGCTGGTTCAATGCAG
R	TGGAAAGGCAGGATTTACCAAC
	ZP3 (5’→3’)
F	GACCCGGGCCAGATACACT
R	CATCTGGGTCCTGCTCAGCTA
